# The effect of a single dose of methylphenidate on attention in children and adolescents with ADHD and comorbid Oppositional Defiant Disorder

**DOI:** 10.1371/journal.pone.0299449

**Published:** 2024-08-12

**Authors:** Barbara D’Aiello, Silvia Di Vara, Pietro De Rossi, Stefano Vicari, Deny Menghini

**Affiliations:** 1 Child and Adolescent Neuropsychiatry Unit, Bambino Gesù Children’s Hospital, IRCCS, Rome, Italy; 2 Department of Human Science, LUMSA University, Rome, Italy; 3 Department of Life Science and Public Health, Catholic University of the Sacred Heart, Rome, Italy; University of Toronto, CANADA

## Abstract

The co-occurrence Oppositional Defiant Disorder (ODD) in children and adolescents with Attention Deficit Hyperactivity Disorder (ADHD) has been associated to difficulties in regulating adverse states, elevated functional impairment, deficits in Executive Functions and high risk for psychopathology. Recent studies have shown that ODD is a negative predictor of a positive response to methylphenidate (MPH) treatment for ADHD symptoms in children and adolescents and that patients with a diagnosis of comorbid ADHD and ODD are less likely to respond favorably to pharmacological treatment with MPH. We conducted a naturalistic study to understand the clinical characteristics of drug-naïve children and adolescents with ADHD that influence the response to MPH by measuring the effect on attention. Specifically, we investigated whether a single dose of MPH differently affects the performance of 53 children and adolescents with ADHD with or without ODD comorbidity. In addition, participant characteristics such as symptom severity, functional impairment, and associated behavioral and emotional symptoms at baseline were examined to better understand what aspects affect the response to MPH. We found that a single dose of MPH improved the attention of children and adolescents with ADHD without ODD more than those with comorbid ADHD and ODD, resulting in reduced reaction times. Our findings indicated that children and adolescents with comorbid ADHD and ODD and those with ADHD alone did not exhibit differences in measures of attention prior to taking MPH, nor in demographic variables (age, intelligence quotient, gender), clinical characteristics related to symptom severity, and adaptive behaviors. However, we observed differences between the two groups in certain behavioral aspects, including the Dysregulation Profile and disruptive behaviors. Assessing symptoms in combination with the presence of ADHD can be beneficial in determining which individuals would derive the greatest benefits from treatment.

## Introduction

Attention Deficit Hyperactivity Disorder (ADHD) is a common and persistent neurodevelopmental disorder emerging during childhood as a mixture of inappropriate and impaired levels of hyperactivity, impulsivity and/or inattention [1].

A study of the Italian pediatric population [2] showed that patients with ADHD and comorbidities are more likely to receive pharmacological treatment, often associated with behavioral therapies. Among co-occurring disorders, patients with ADHD and Oppositional Defiant Disorder (ODD) most frequently receive treatment with methylphenidate (MPH) the first-choice treatment for ADHD [3,4]. In fact, ODD symptoms may exacerbate clinical severity and worsen long-term outcomes of ADHD [5], produce greater difficulties in daily coping, at school, and in other life settings. Accordingly, patients with ADHD and ODD in comorbidity show reduced levels of well-being, and greater social and individual problems than their non-comorbid counterparts [6]. It was observed [7] that differences in Executive Functions (EF) can predict severity of ODD symptoms to varying degrees. More specifically, better working memory abilities and attention, assessed in children at the ages of 3 and 4, were linked to lower negative affect scores in ODD at age 6 during school age, even when controlling for ADHD symptoms [7]. Deficits in EF could lead to ineffective problem-solving and an inability to adapt behavior to different situations, ultimately resulting in difficulties in regulating emotions [8]. The EF have been extensively studied in patients with ADHD, whereas fewer studies have investigated EF in patients with ODD, with mixed results. Some studies found that children with ODD as well as those with comorbid ADHD and ODD, had deficits in EF including inhibitory control [9,10], attention [11], and working memory [12]. Other studies have concluded that relations between ODD and EF are exclusively the result of comorbid ADHD [13,14].

There is evidence that MPH, significantly improved EF and that the effect on EF has been considered one of the indicators of response to MPH [13–15]: when MPH dosing is optimized the performance on EF of most patients with ADHD reaches the level of control children [16]. Specifically, inhibitory control [15,17–19], visual-spatial working memory [20], and sustained and selective attention [21] of children and adolescents with ADHD have been improved by MPH [15,22–24]. The improvement found on EF after MPH treatment was also observed after a single dose with notable effects during tasks of attention [25,26] and other tasks such as temporal processing [27] and inhibitory control [25,27]. Improvement after a single dose of MPH has been identified also as a predictor of MPH response after 4 weeks [28] and 1 year of treatment [29].

Overall, although the effects of MPH on ADHD symptoms are proven, there is currently no definitive guidance on how patients with comorbidities, such as ODD, respond to MPH [30] and which is the best pharmacological management of comorbidities [31]. We know that the comorbidity associated with ADHD might play a role in modulating the response to MPH [13]. However, while some studies in ADHD have found that comorbidity is associated with a worse response to MPH [13,32–35], other studies have not confirmed an association between MPH response and comorbidity [36,37]. In order to understand the clinical characteristics of children and adolescents with ADHD that influenced the response to MPH, we conducted a naturalistic study in drug-naïve patients with ADHD. Specifically, we tested whether a single dose of MPH differentially improved attention of patients with ADHD with or without ODD.

## Materials and method

### Participants

Fifty-three children and adolescents with combined presentation of ADHD (46 males and 7 females), who received MPH for the first time at the Child and Adolescent Neuropsychiatry Unit of the Bambino Gesù Children’s Hospital, were included in the study and divided into two subgroups.

The first subgroup consisted of 26 participants with comorbid ADHD and ODD (Table 1), who met the following inclusion criteria: 1) the presence of an ADHD diagnosis (combined presentation) accordingly to the Diagnostic and Statistical Manual of Mental Disorders, Fifth Edition—DSM-5 [1]; 2) the presence of an ODD diagnosis defined by a frequent and persistent pattern of irritable and angry mood, vindictiveness and developmentally inappropriate, negativistic, defiant, and disobedient behavior toward authority figures accordingly to the DSM-5 [1]; 3) having carried out at least 6 months of psychosocial and psycho-behavioral interventions; 4) needing drug treatment for the severity of the ADHD symptoms; 5) drug naïve; 6) an intelligence quotient (IQ) of 80 or higher; 7) normal or corrected-to-normal vision; 8) caregiver consent to drug treatment.

**Table 1:** Demographic information of subgroup with comorbid ADHD and Oppositional Defiant Disorder and subgroup with ADHD without Oppositional Defiant Disorder.

	Comorbid ADHD and ODD	ADHD without ODD
**Males/Females**	24/2	22/5
**Age (mean ± SD)**	10.3 (2.7)	11.0 (2.6)
**IQ (mean ± SD)**	101.9 (10.7)	101.4 (11.6)

**Fig 1:**
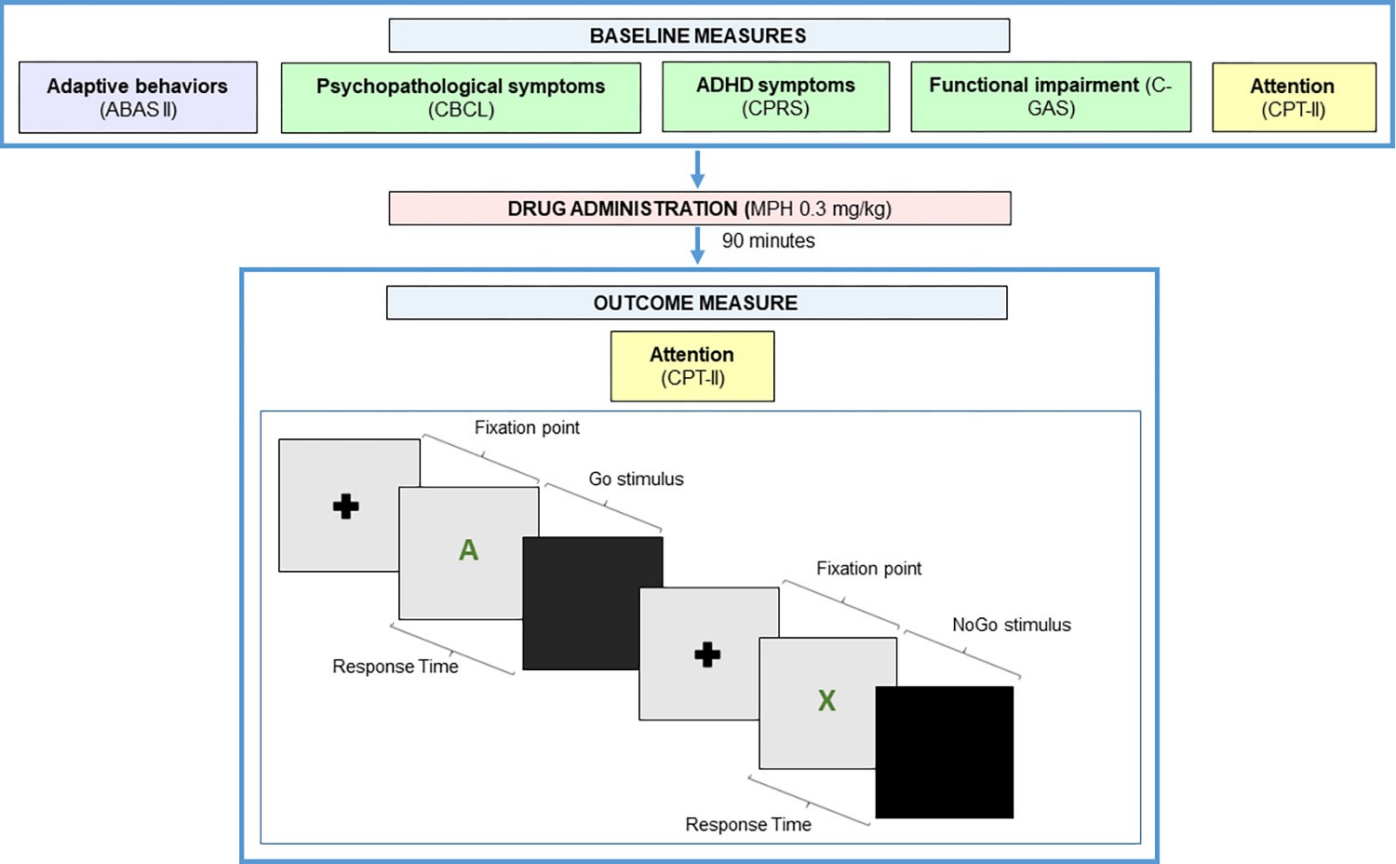
Overview of the study design. Following baseline assessment, each participant repeated the Continuous Performing Test II [38] after a single dose of MPH administration.

After assessing the presence of ADHD and ODD, we evaluated adaptive behaviors, severity of ADHD symptoms, behavioral and emotional symptoms at baseline (T0). Attention was tested at T0 and retested after a single dose of MPH administration (T1) using the Continuous Performance Test II [38] and was considered the outcome measure (see Fig 1).

The second subgroup of children and adolescents consisted of 27 participants with ADHD without ODD (Table 1), and they met the following inclusion criteria: 1) the presence of an ADHD diagnosis (combined presentation) accordingly to the Diagnostic and Statistical Manual of Mental Disorders, Fifth Edition—DSM-5 [1]; 2) the absence of an ODD diagnosis; 3) having carried out at least 6 months of psychosocial and psycho-behavioral interventions; 4) needing drug treatment for the severity of the ADHD symptoms; 5) drug naïve; 6) an IQ of 80 or higher; 7) normal or corrected-to-normal vision; 8) caregiver consent to drug treatment.

The IQ was measured by the Wechsler Intelligence Scale for Children [39], Colored Progressive Matrices [39,40] or Standard Progressive Matrices [41]. For both subgroups exclusion criteria were 1) concomitant drug treatment; 2) the presence of other neurodevelopmental disorders (i.e., autism spectrum disorders) or other psychiatric disorders (i.e., bipolar disorders, schizophrenia spectrum disorders, or adjustment disorder) as comorbid conditions; 3) a history of neurological or medical or genetic conditions; and 4) a basal medical condition (i.e., heart, kidney, or liver diseases) that may exclude the possibility to administer MPH.

### Assessment

The diagnosis of ADHD, ODD and psychiatric conditions were based on developmental history, extensive clinical examination and the administration of the Schedule for Affective Disorders and Schizophrenia for School Aged Children Present and Lifetime Version DSM-5 [42], a semi-structured interview that assesses the presence of psychopathological disorders according to DSM-5 classification [1].

The level of impairment of the person’s overall functioning was evaluated by using the Children Global Assessment Scale [43]. According to the cut-off thresholds of the scale, scores from 100 to 60 indicate an adequate functioning, scores from 60 to 40 show the presence of evident problems, scores from 40 to 30 indicate the presence of serious problems and scores lower than 30 reveal serious/extreme compromises.

Severity of ADHD symptoms was assessed by considering two Global Index subscales (Global Index Restless/Impulsive and Global Index Emotional Liability) and two DSM-IV subscales (Inattentive, Hyperactive/Impulsive) of Conners’ Parent Rating Scale [44]. Conners’ Parent Rating Scale contains 80 items, each of which is required for a response according to Likert scale of 4 levels (from 0, which indicates a behavior not present or detectable rarely subject, to 3, indicating the presence of persistent or very frequent behavior). According to the cut-off thresholds of Conners’ Parent Rating Scale manual, the T-scores cut off for significance is >70 (very high) while T-scores of 60 to 70 are considered high average or elevated.

Behavioral and emotional symptoms were assessed by the parent questionnaire Child Behavior Checklist [45]. Child Behavior Checklist requires parents to evaluate child’s behavior and emotion during the preceding 6 months on a 3-point Likert scale for each of the 113 items (0 = Not True; 1 = Somewhat or Sometimes True; 2 = Very True or Often True). We considered only Syndrome Scales (Anxious/Depressed, Withdrawn/Depressed, Somatic Complaints, Social Problems, Thought Problems, Attention Problems, Rule-Breaking Behavior, and Aggressive Behavior) to avoid overlapping items. According to the cut-off thresholds of Achenbach and Rescorla [45], T-scores > 69 are classified as clinically relevant, T-scores between 65 and 69 are classified as borderline, and T-scores < 65 indicate nonclinical symptoms. The Child Behavior Checklist Dysregulation Profile was calculated by considering the sum of T-scores of three Syndrome Scales: Anxious/Depressed, Attention Problems, and Aggressive Behavior. Scores ≥ 210 are considered clinical, scores between 180 and 209 are in the borderline range, and scores ≤ 179 are not clinically significant [46–48].

Adaptive behaviors were assessed using the Adaptive Behavior Assessment System-Second Edition questionnaire [49] completed by parents. Responses to questions are rated on a 4-point Likert scale (0 = is not able; 1 = never or almost never; 2 = sometimes when needed; and 3 = always or almost always). According to the cut-off thresholds, we considered composite scores (with mean of 100 and standard deviation of 15) from 3 different domains: Conceptual, Social, and Practical.

Continuous Performing Test II [38] was used to measure attention. Continuous Performing Test II is a task-oriented computerized assessment of composed by 360 letters, presented one at a time for approximately 250 ms each, which are presented in the standard format of 18 sub-blocks of 20 trials each. The blocks differ in the interstimulus intervals (ISI) between letter presentations which last 1, 2, or 4 s. ISI are randomized between blocks so that all three ISI conditions occur every three blocks. Transition from one block to the next is unannounced and occurs without delay. The participants were instructed to press the spacebar when any letter except the letter “X” appeared on the screen. The percentage of trials when letters other than “X” appear is 90% across all ISI blocks. No-Go trials occur when an “X” is presented. All participants had a 3-min practice session prior to starting the Continuous Performing Test II, to reduce the effect of familiarity stemming from repeated tests. Accuracy, Reaction Times (RTs) in millisecond, and variability of RTs (VRTs) in millisecond were recorded at T0 and T1 and converted to z-scores by considering the means and standard deviations of all participants together.

### Medication

MPH is the first-line medication for children and adolescents with ADHD according to National Institute for Health and Care Excellence and Agenzia Italiana del Farmaco guidelines. Before taking MPH, all patients who were eligible for treatment underwent an electrocardiogram examination with calculation of the corrected QT interval, and blood tests to rule out any other medical condition that might be associated with ADHD or potentially mimic ADHD symptoms (e.g., thyroiditis). All participants performed Continuous Performing Test II one day before drug administration (T0) and 90 minutes after administration of 0.3 mg/kg of the short-acting MPH preparation Ritalin© (T1).

### Statistical analyses

Chi-square analysis was conducted to verify that subgroup with comorbid ADHD and ODD and subgroup with ADHD without ODD did not differ by gender. Student’s t-tests were used to compare age, IQ, Children Global Assessment Scale and the Child Behavior Checklist Dysregulation Profile of subgroup with comorbid ADHD and ODD and subgroup with ADHD without ODD (Bonferroni’s corrected p-value for 4 multiple comparisons = 0.05/4). Multivariate Analysis of Variance was used to compare subgroup with comorbid ADHD and ODD and subgroup with ADHD without ODD on adaptive measures (Conceptual, Social, Practical Domain of Adaptive Behavior Assessment System-Second Edition), behavioral and emotional symptoms (Syndrome Scales of Child Behavior Checklist), and ADHD severity symptoms (4 subscales of Conners’ Parent Rating Scale). Post-hoc analyses were conducted by using Tukey HSD test. A Repeated Measures Analysis Of Variance) with Group (comorbid ADHD and ODD vs ADHD without ODD) as between-subject factor and Time (T0, T1) as within-subjects factor was run to verify the effect of MPH on z-scores of Continuous Performing Test II (Accuracy, RTs, and VRTs). Partial eta squares (ηp2) were reported as measures of effect size. Post-hoc analyses were conducted by using Tukey HSD test. The statistical software SPSS Version 22 (IBM Corporation, Armonk, NY, USA, 2017) was used for analyses.

### Power and sample size considerations

We performed a priori power analysis using G*Power 3.1.9 [50] to ascertain the appropriate sample size. The estimated result is obtained on the assumption that participants under the influence of MPH would exhibit improved performance in attentional tasks compared to the baseline.

In line with the meta-analysis conducted by Tamminga and colleagues [51], we set the effect size at 0.42 [ES = 0.42, p < 0.0001, 95% CI 0.26–0.59], with a significance level of p < 0.05. The desired power was set at 0.80, considering 2 groups and 3 measurements (CPT-II RTs, VRTs, Accuracy) in the analysis. The critical F-value required to achieve the desired power was set at 3.16. To test our hypothesis, we determined that a sample size of 58 subjects was needed.

## Results

### Demographic characteristics

Subgroup with comorbid ADHD and ODD and subgroup with ADHD without did not differ for age (t _(51)_ = 0.99, *p* = 0.32), IQ (t _(51)_ = -0.16, *p* = 0.87), gender (χ^2^_(1)_ = 1.48, *p* = 0.22), and global functioning—Children Global Assessment Scale scores (comorbid ADHD and ODD: 48.34 ± 7.37); ADHD without ODD: 50.85 ± 6.65); t _(51)_ = 1.29, *p* = 0.20).

### Adaptive behaviors

The two subgroups did not differ (F _(1, 51)_ = 1.92, *p* = 0.17) on adaptive subscales (Conceptual, Social, Practical Domain) of Adaptive Behavior Assessment System-Second Edition. Also, the Group x Adaptive Subscale interaction (F _(2,102)_ = 0.71, *p* = 0.49) was not significant (for details see S1 Table). However, Adaptive Subscale effect was significant (F _(2, 102)_ = 6.81, *p* = 0.001). Post-hoc analyses conducted by using Tukey HSD test on Adaptive Subscales, independently of the subgroup, showed lower scores on Practical Domain (65.72 ± 2.32) than on Conceptual Domain (72.85 ± 1.70, *p* = 0.001) and than on Social Domain (70.68 ± 2.12, *p* = 0.03). Scores of Conceptual Domain and Social Domain subscales did not differ (*p* = 0.50).

### Behavioral and emotional symptoms

The Group effect on Syndrome Scales of Child Behavior Checklist was significant (F _(1, 51)_ = 21.59, *p* < 0.001, η_p_^2^ = 0.29) with higher scores of subgroups with comorbid ADHD and ODD (69.10 ± 0.97) than subgroup with ADHD without ODD (62.65 ± 0.97). The Syndrome Scales effect (F _(7, 357)_ = 12.93, *p* < 0.001, η_p_^2^ = 0.20) and the Group x Syndrome Scales interaction (F _(7, 357)_ = 2.58, *p* = 0.013, η_p_^2^ = 0.04) were also significant. The subgroup with comorbid ADHD and ODD (70.07 ± 7.36) had significantly higher scores than subgroup with ADHD without ODD (59.14 ± 6.50) in Rule-breaking Behavior (*p* < 0.001). Moreover, subgroup with comorbid ADHD and ODD (74.30 ± 9.69) showed higher scores than subgroup with ADHD without ODD (63.33 ± 9.22) in Aggressive Behavior (*p* < 0.001). No differences were found in other Syndrome Scales (for details see S2 Table).

Subgroup with comorbid ADHD and ODD had significantly higher scores (215.53 ± 23.32) than the subgroup with ADHD without ODD (196.14 ± 19.26) in the Child Behavior Checklist Dysregulation Profile (t _(51)_ = -3.30, *p* = 0.001).

Considering severity of ADHD symptoms, the Group effect on Conners’ Parent Rating Scale subscales (Restless/Impulsive, Emotional Liability, Inattentive, Hyperactive/Impulsive) was significant (F _(1, 51)_ = 4.16, *p* = 0.04, η_p_^2^ = 0.07) with higher scores for subgroup with comorbid ADHD and ODD than subgroup with ADHD without ODD. The Conners’ Parent Rating Scale effect (F _(3, 153)_ = 37.01, *p* < 0.001, η_p_^2^ = 0.42) and the Group x Conners’ Parent Rating Scale subscales interaction were also significant (F _(3, 153)_ = 4.53, *p* = 0.004, η_p_^2^ = 0.08). A significant difference (*p* = 0.002) between subgroup with comorbid ADHD and ODD and subgroup with ADHD without ODD in Global Index Restless/Impulsive subscales was found, with higher scores for subgroup with comorbid ADHD and ODD (71.53 ± 2.88) than subgroup with ADHD without ODD (51.58 ± 2.82). No differences were found in other subscales (for Mean and SD of others Conners’ Parent Rating Scale subscales see S3 Table).

### Attention

Regarding Continuous Performing Test II measures (Accuracy, RTs, and VRTs), the Group effect was significant (F _(1, 51)_ = 9.50, *p* = 0.003, η_p_^2^ = 0.15) with lower z-scores for subgroup with comorbid ADHD and ODD (-0.26 ± 0.11) compared to subgroup with ADHD without ODD (0.25 ± 0.11). The Group x Time interaction was also significant (F _(1, 51)_ = 5.35, *p* = 0.02, η_p_^2^ = 0.09), with subgroup with ADHD without ODD presenting a significant improvement (*p* = 0.05) between T0 and T1 and subgroup with ADHD without ODD did not (*p* = 0.06). Also, the Group x Time x Continuous Performing Test II measures interaction (F _(2, 102)_ = 6.29, *p* = 0.002, η_p_^2^ = 0.10) was significant (Table 2), with lower RTs at T1 (*p* = 0.02) in subgroup with ADHD without ODD (z-score: 0.46 ± 0.66) compared to subgroup with comorbid ADHD and ODD (z-score: -0.48 ± 1.06). No significant difference was found between subgroup with ADHD without ODD (z-score: 0.08 ± 0.98) and subgroup with comorbid ADHD and ODD (z-score: -0.09 ± 1.02) in RTs at T0 (*p* = 0.99). Moreover, the Group x Continuous Performing Test II measures interaction (F _(2, 102)_ = 0.06, *p* = 0.94) was not significant.

**Table 2:** Mean and standard deviation of raw scores of continuous performing test II measures in subgroup with comorbid ADHD and Oppositional Defiant Disorder and subgroup with ADHD without Oppositional Defiant Disorder.

	T0		T1	
	Comorbid ADHD and ODD Mean (SD)	ADHD without ODD Mean (SD)	Comorbid ADHD and ODD Mean (SD)	ADHD without ODD Mean (SD)
**Accuracy**	76.98 (33.51)	90.67 (18.89)	80.31 (34.88)	92.76 (18.97)
**RTs (ms)**	452.05 (96.49)	434.95 (92.06)	475.91 (86.08)	398.86 (53.73)
**VRTs (ms)**	303.25 (158.80)	253.21 (144.36)	250.61 (125.32)	167.85 (111.67)

## Discussion

It is estimated that about half of children with ADHD also meet the diagnostic criteria for ODD [52]. When the two disorders co-occur, the age of onset of ADHD symptoms is earlier, the severity of symptoms is greater, and the risk of developing further psychiatric disorders is higher [53].

The present study examined whether the comorbidity of ADHD with ODD could influence the response to a single dose of MPH, considering measures of attention, given that an association between inattention and symptom severity was found in both ADHD and ODD [52].

Our results showed that children and adolescents with ADHD without ODD responded better to MPH than those with ODD, as documented by the positive effect obtained on RTs of Continuous Performing Test II. We can interpret our results by taking into account the cognitive processes and the activity of the brain areas involved in this task. Continuous Performing Test II assesses different cognitive processes such as maintenance and updating, response selection and inhibition/suppression, and performance monitoring [38,54]. It has been shown [55–57] that patients with ADHD exhibited atypical activation in the lateral prefrontal cortex, striato-thalamic regions, anterior cingulate cortex and cerebellum. Although brain abnormalities in ADHD and ODD often overlapped [58], neuroimaging studies [59] have documented greater reduction of volume or activity in the bilateral amygdala, right striatum (including caudate, putamen, and globe pallidum), bilateral insula, and ventromedial circuits in patients with comorbid ADHD and ODD than in patients with ADHD without ODD, who were more impaired in ventrolateral circuits. Even when ADHD was not in comorbidity and patients presented only disruptive behaviors such as ODD, functional neural abnormalities have been confirmed in the amygdala and ventromedial prefrontal cortex [56–62]. The literature has documented that MPH significantly upregulated lateral prefrontal cortex and striatal circuits during attention tasks (such as Continuous Performing Test II) compared to medial frontal regions (and temporal) in patients with ADHD [51]. It could be hypothesized that our patients with comorbid ADHD and ODD were less responsive to the effect of MPH because they had greater impairment of ventromedial circuits and consequently performance at Continuous Performing Test II improved less than patients with ADHD without ODD.

Our results were in line with previous studies that have shown in ADHD with higher ODD symptoms reduced effects of MPH [63,64]. Specifically, a recent study analyzed the behavioral and neuropsychological symptoms of 518 children and adolescents with ADHD at baseline that could predict the response to MPH [13]. Results showed that ODD and other comorbid symptoms, such as depression, were associated with a worse clinical response to MPH in patients with ADHD.

It should be noted that the children and adolescents with comorbid ADHD and ODD and with ADHD without ODD included in our study did not differ in measures of attention before taking MPH, nor in other variables (age, IQ, gender), clinical characteristics of symptom severity (Children Global Assessment Scale) and adaptive behaviors (Adaptive Behavior Assessment System-Second Edition). Therefore, the effect given by MPH on Continuous Performing Test II only in the subgroup with ADHD without ODD cannot be explained by the differences at baseline in the two groups, supporting the suggestion that it is indeed due to MPH. However, our results showed differences between the two groups in some behavioral aspects, such as the Child Behavior Checklist Dysregulation Profile and disruptive behaviors (Aggressive Behavior and Rule Breaking Behavior subscales) assessed by Syndrome Scales of Child Behavior Checklist. Our findings are in line with those of previous studies that have found higher levels of reactive and proactive aggression [65,66] and more prominent psychopathy traits [67] in children with comorbid ADHD and ODD. Similarly with regard to the Child Behavior Checklist Dysregulation Profile, which is characterized by low frustration tolerance, impatience, and ease of emotional reaction [68], this trait has been found to be highly prevalent in disruptive disorders such as ODD [69].

Assessing the presence of ODD in patients with ADHD before MPH administration could help clinicians to identify individuals who respond better to the medication or, conversely, to hypothesize alternative treatments in the presence of ODD. First-line treatments for ODD are behavioural family therapies while medications are used as additional treatments for severe or treatment-resistant children [70]. Although children with ADHD and ODD are usually treated with stimulant medication [71], other drugs with different evidence of efficacy are also used to treat oppositional symptoms, such as clonidine [72], guanfacine [73], atomoxetine [74] and risperidone [75]. For children with ADHD with co-morbid ODD, MPH and risperidone appear to be effective on aggressive behaviours, but only MPH seems to have an effect on ADHD symptoms [31] and in any case is more effective than antipsychotics or mood stabilizers in reducing aggression [75].

Our results on the Conners’ Parent Rating Scale subscales showed that the two groups did not differ in the severity of ADHD symptoms, except for impulsivity (Global Index Restless/Impulsive subscales), which was higher in patients with comorbid ADHD and ODD than in patients with ADHD without ODD. Consistent with our result, it has been observed that children and adolescent with ADHD with significant impulsivity are more at risk of exhibiting externalizing behaviors such as oppositionality, aggression and conduct disorder than children with ADHD without significant impulsivity [35].

It should be noted that emotional and behavioral symptoms are moderated by relationships and context in fact it has been observed that family processes mitigate the association between ODD and impulsiveness [76]. For these reasons, it is important to combine drug therapy such as MPH or risperidone with psycho-educational interventions for parents and teachers [54].

Another factor to consider in interpreting our results is the possibility of a slightly higher dose-effect range for children with comorbid ADHD and ODD. The observed outcome approached significance and could be achievable with slightly increased doses. Current guidelines [77] advocate for tailoring MPH dosages to individual patient needs, recognizing various factors influencing dose optimization. These factors include genetic variability, patient weight, age, gender, drug-induced tolerance, and interactions with other medications or medical conditions [78]. Notably, a study identified a dose-response relationship with MPH in children with ADHD combined presentation, but this relationship was absent in those with ADHD predominantly inattentive presentation [79]. Thus, it is plausible to hypothesize that patients with more complex symptomatology may necessitate a higher dosage.

In conclusion, our results showed that after a single dose of MPH, children and adolescents with ADHD without ODD showed a significant improvement in attention, in contrast to patients with comorbid ADHD and ODD. Our results highlight the importance of investigating the comorbidities in defining the therapy to be administered to children with ADHD.

Developing studies that pay attention to comorbidities, which are often frequent in patients with ADHD, can help clinicians identify who is most likely to benefit from MPH treatment, thereby improving future drug indications for children and adolescents with ADHD.

## Limitations

Our study has some limitations.

The first limitation is the absence of a control group, which would have allowed us to confirm the attention deficit typically found in patients with ADHD in our study.

A second limitation is that the present study is not longitudinal, and it is not possible to make long-term inferences about how improvement in attention in children and adolescents with ADHD without ODD can be maintained over time with MPH treatment.

Another limitation is that we need to understand whether the effects of MPH that we found on attention also generalize to the behavioural manifestations of ADHD, using clinical measures.

Moreover, it’s important to note that this is a naturalistic study which could introduce bias into the results.

Additionally, there was a lack of gender diversity among the participants, which could impact the generalizability of the findings to a more diverse population. Furthermore, a larger sample would strengthen the significance of our results. The outcomes derived from our study’s small sample size, which did not reach the required participant number of 58 as specified by the power analysis, may be particularly vulnerable to random variations, diminishing their reliability. Consequently, it is imperative to replicate the study with a more substantial sample to confirm whether the absence of ODD genuinely correlates with improved efficacy of MPH drug therapy.

Furthermore, response to drug treatment should be monitored through longitudinal studies.

It would be useful to consider other comorbidities beyond ODD and to include additional treatment outcomes, such as symptom reduction in various life contexts or specific neuropsychological measures (i.e., inhibition).

## Supporting information

S1 Table Mean and standard deviation of adaptive behaviors (from Adaptive Behavior Assessment System-Second Edition) in subgroup with comorbid ADHD and Oppositional Defiant Disorder and subgroup with ADHD without Oppositional Defiant Disorder. (DOCX)

S2 Table Comparisons between subgroup with comorbid ADHD and ODD and subgroup with ADHD without Oppositional Defiant Disorder on behavioral and emotional symptoms (from Child Behavior Checklist). (DOCX)

S3 Table Comparisons between subgroup with comorbid ADHD and ODD and subgroup with ADHD without Oppositional Defiant Disorder on severity of ADHD symptoms (from Conners’ Parent Rating Scale). (DOCX)
