# Supplementary material for: The effect of a single dose of methylphenidate on attention in children and adolescents with ADHD and comorbid Oppositional Defiant Disorder
Source: PLoS One. 2024 Aug 12;19(8):e0299449. doi: 10.1371/journal.pone.0299449 (PMC11318934; doi:10.1371/journal.pone.0299449)
Supplement: S1 Table — (DOCX) [file pone.0299449.s001.docx]

*Supplementary Information*

**Table S1. Mean and standard deviation of adaptive behaviors (form Adaptive Behavior Assessment System-Second Edition) in subgroup with comorbid ADHD and ODD and subgroup with ADHD without ODD**

|  | Comorbid ADHD and ODDMean (SD) | ADHD without ODDMean (SD) |
| --- | --- | --- |
| Conceptual Domain | 71.74 (2.38) | 73.96 (2.43) |
| Social Domain | 67.25 (2.97) | 74.11 (3.03) |
| Practical Domain | 63.11 (3.25) | 68.34 (3.31) |
