# Supplementary material for: The effect of a single dose of methylphenidate on attention in children and adolescents with ADHD and comorbid Oppositional Defiant Disorder
Source: PLoS One. 2024 Aug 12;19(8):e0299449. doi: 10.1371/journal.pone.0299449 (PMC11318934; doi:10.1371/journal.pone.0299449)
Supplement: S2 Table — (DOCX) [file pone.0299449.s002.docx]

*Supplementary Information*

**Table S2. Comparisons between subgroup with comorbid ADHD and ODD and subgroup with ADHD without ODD on behavioral and emotional symptoms (from Child Behavior Checklist)**

|  | Comorbid ADHD and ODD **Mean (SD)** | ADHD without ODD **Mean (SD)** | **p-value**  **Tukey HSD test** |
| --- | --- | --- | --- |
| **Anxious/Depressed** | 66.19 (8.55) | 62.92 (9.40) | 0.98 |
| **Withdrawn/Depressed** | 66.96 (6.98) | 59.22 (6.99) | 0.055 |
| **Somatic Complaints** | 63.73 (9.58) | 59.92 (7.46) | 0.95 |
| **Social Problems** | 67.65 (7.33) | 63.22 (7.12) | 0.85 |
| **Thought Problems** | 68.84 (8.82) | 62.85 (7.31) | 0.37 |
| **Attention Problems** | 75.03 (11.52) | 70.62 (7.30) | 0.86 |
| **Rule-breaking Behavior** | 70.07 (7.36) | 59.14 (6.50) | 0.0002 |
| **Aggressive Behavior** | 74.30 (9.69) | 63.33 (9.22) | 0.0001 |
