# Supplementary material for: The effect of a single dose of methylphenidate on attention in children and adolescents with ADHD and comorbid Oppositional Defiant Disorder
Source: PLoS One. 2024 Aug 12;19(8):e0299449. doi: 10.1371/journal.pone.0299449 (PMC11318934; doi:10.1371/journal.pone.0299449)
Supplement: S3 Table — (DOCX) [file pone.0299449.s003.docx]

*Supplementary Information*

**Table S3. Comparisons between subgroup with comorbid ADHD and ODD and subgroup with ADHD without ODD on severity of ADHD symptoms (from Conners' Parent Rating Scale)**

|  | Comorbid ADHD and ODDMean (SD) | ADHD without ODDMean (SD) | p-valueTukey HSD test |
| --- | --- | --- | --- |
| Restless/Impulsive | 71.53 (2.9) | 58.5 (2.8) | 0.002 |
| Emotional Liability | 80.61 (2.3) | 76.4 (2.3) | 0.92 |
| Inattentive | 83.11 (1.7) | 81.5 (1.6) | 0.99 |
| Hyperactive/Impulsive | 78.8 (2.5) | 76.3 (2.5) | 0.99 |
